# Anionic Lipids: Determinants of Binding Cytotoxins from Snake Venom on the Surface of Cell Membranes

**Published:** 2010-07

**Authors:** A.G. Konshina, I.A. Boldyrev, A.V. Omelkov, Yu.N. Utkin, R.G. Efremov

**Affiliations:** Shemyakin and Ovchinnikov Institute of Bioorganic Chemistry, Russian Academy of Sciences; Faculty of Technology of Organic Substances and Chemical Pharmaceutical Compounds, Mendeleev University of Chemical

**Keywords:** cytotoxins, phosphatidylserine, lipid membranes, molecular docking, fluorescent spectroscopy

## Abstract

The cytotoxic properties of cytotoxins (CTs) from snake venom are mediated by their interaction with the cell membrane. The hydrophobic pattern containing the tips of loops I–III and flanked by polar residues is known to be a membrane–binding motif of CTs. However, this is not enough to explain the difference in activity among various CTs which are similar in sequence and in 3D structure. The mechanism of further CT–membrane interaction leading to pore formation and cell death still remains unknown. Published experimental data on the specific interaction between CT and low molecular weight anionic components (sulphatide) of the bilayer point to the existence of corresponding ligand binding sites on the surface of toxin molecules. In this work we study the membrane–lytic properties of CT I, CT II (*Naja oxiana*), and Ct 4 (*Naja kaouthia*), which belong to different structural and functional types (P– and S–type) of CTs, by measuring the intensity of a fluorescent dye, calcein released from liposomes containing a phosphatidylserine (PS) lipid as an anionic component. Using molecular docking simulations, we find and characterize three sites in CT molecules that can potentially bind the PS polar head. Based on the data obtained, we suggest a hypothesis that CTs can specifically interact with one or more of the anionic lipids (in particular, with PS) contained in the membrane, thus facilitating the interaction between CTs and the lipid bilayer of a cell membrane.

## INTRODUCTION

One of the features of cytotoxins (CTs, sometimes called cardiotoxins) from snake venom is their toxicity to cells of various types [1]. CTs are small basic proteins from the three–finger toxin group. The secondary structure of CTs consists of two β –sheets that have 2 and 3 antiparallel β –strands and is stabilized with 4 disulphide bonds. The major differences between the amino acid sequence and the 3D structure of members of this large CT group occur in irregular loop segments, primarily in loops I and II. Depending on the presence of the conservative residues S28 (S–type) or P30 (P–type) in loop II, CTs exhibit different functional activities [2]. For a long time, CTs were believed to interact with the cell membrane in a nonspecific way. P–type CTs interact with the bilayer more actively than S–type CTs due to the more hydrophobic loop II, resulting in an extended hydrophobic region on the toxin ’ s surface formed by the hydrophobic tips of the three loops. The amphiphilic properties of CT molecules (the hydrophobic ends of loops I–III are flanked with cationic residues) enable their embedding into the lipid bilayer, creating porous defects in the membrane [3–6] and leading to the cell death. Several models of the mechanism of bilayer damage by CT and lysis have been proposed [7]. It was shown later that some CTs, for instance the CT А3 ( * Naja atra * ), can penetrate inside the cell and interact with cell organelles, mitochondria [8], and lysosomes [9].

The biological activity of CTs is very diverse. In particular, they can modify the function of various membrane proteins, such as protein kinase C (PKC), Na^+^, K^+^–ATPase , and integrins. Therefore, the hypotheses about the specific CT targets and molecular mechanisms of toxin–membrane interaction are of interest and have been actively discussed [7, 10–12].

Based on NMR and X–ray data, a search for potential CT targets revealed a series of anionic low molecular weight ligands (heparin–derived oligosaccharides, ATP derivatives, and sulphatide) that interact with certain sites on the toxin’s surface [13–17]. Fluorescent spectroscopy studies on liposomes of various composition combined with * in vivo * experiments using monoclonal antibodies indicate a specific CT target—a polar head of a glycolipid sulphatide (hSGC)—located on the surface of a rat’s cardiomyocyte membrane, which mediates the toxic activity of CT А3 [17]. Taking into account the fact that CTs affect various cell types and that toxins effectively lyse model membranes containing no glycolipids [5, 18, 19], one can expect CT to be able to interact nonspecifically with other anionic membrane lipids, including the most common one, phosphatidylserine (PS). Although this lipid is normally localized in the inner leaflet of the cell membrane, when the bilayer is damaged in certain pathologies, PS is present in the outer leaflet and can be accessible to CT molecules. Some experimental data indirectly support the possibility that there is competition for a site on the membrane’s surface between CT and other PS–binding molecules (PKC, thionin) [10, 20, 21].

In this work we assumed that PS is a specific target mediating CT cell toxicity and studied the interaction of S–type (CT I from *Naja oxiana*) and P–type (CT II from *Naja oxiana* and CT 4 from *Naja kaouthia*) cytotoxins with palmitoyl oleyl phosphatidylcholine (POPC) membranes, including those containing PS and SGC. It should be noted that the primary structure of CT 4 (*N. kaouthia*) is identical to that of CT А3 (*N. atra* ). Using fluorescent spectroscopy, we studied the lytic activity of the selected CTs on liposomes of various composition. In order to identify sites in a CT molecule capable of binding PS, we performed molecular docking simulations of the PS polar head using 3D models of toxins active with respect to PS–containing liposomes. Based on the results obtained, we hypothesized that toxins interact with the bilayer * via * a two–stage specific mechanism, the molecular determinants of which are the polar heads of the anionic lipids, which interact with specific CT sites.

## EXPERIMENT

**Fluorescent Spectroscopy: CT Lytic Activity on PS–Containing Liposomes of Various Composition**. CT I and CT II were extracted from * N. oxiana* venom following the technique described in [22;]. CT 4 was extracted from * N. k* *aouthia * venom using the technique proposed in [23] and refined by reverse phase chromatography. The toxin’s structure was confirmed by mass–spectrometry using peptide mapping after the trypsin hydrolysis of the reduced pyridylethylated toxin derivative. Liposomes were prepared using SGC and PS extracted from bovine brain in the Laboratory of Lipid Chemistry, Institute of Bioorganic Chemistry, Russian Academy of Sciences (IBC RAS), and synthetic POPC (Avanti Polar Lipids, United States). Liposomes of the following composition were prepared: POPC, POPC /PS5 %, POPC /PS2 0%, POPC /PS3 5%, POPC /PS50 %, POPC /PS7 0%, POPC /SGC5 %, and POPC /SGC50 %. Lipid solutions in chloroform/methanol (1:1) at appropriate concentrations were mixed, evaporated, and dried in vacuum. The lipid films were hydrated with a buffer containing 5 0 m M tris–НСl (pH 7.8), 30 mM NaCl, 4 mM EDTA, and 100 mM calcein. The suspension was incubated for several hours, subjected to 10 freeze/thaw cycles, and then forced through a 100–nm polycarbonate filter (NucleoPore, USA) 20 times using a mini–extruder (Avanti Polar Lipids, United States). The external dye was eliminated by gel filtration using a Sepharose СL–4B column equilibrated with a buffer containing 50 mM tris–НСl (pH 7.8), 11 0 mM NaCl, and 4 mM EDTA. In addition, the activity of CT 4 with respect to SGC–containing liposomes (POPC/SGC50%) was measured under the conditions described in [6] (buffer: 10 mM tris–НСl (p H 7 .4), 75 mM NaCl; 50 mM 6–carboxyfluorescein (6–CF) as fluorophore; lipid/protein ratio (L/P) ~62). The level of liposome membrane damage by CTs was estimated from spectrophotometric measurements of the amount of fluorophore released from inside the liposomes, * I * , upon adding an aliquot of the toxin solution into the quartz cuvette with the liposome sample. The value of * I * (%) was calculated using the following equation:

*I * = 100 x ( * F – F_0_* ) / ( *F_t_ – F_0_* ),

where *F_0_* is the fluorescent signal from liposomes without the toxin (background level); * F * is the fluorescent signal from liposomes in the presence of the toxin; and * F_t_* is the fluorescent signal after the liposomes have been destroyed with a detergent (Triton X–100).

The L/P ratio in all experiments was 100, unless otherwise stated. The fluorescent signal was measured at 517 nm, with excitation at 494 nm, and the spectral slit width for excitation and emission was 3 and 5 nm, respectively. For each experiment, the kinetics of the fluorescent signal was monitored (for about 40 min) and the final measurements were taken when * I * reached a plateau. For reproducibility control, all measurements were repeated 2–3 times. All measurements were performed at room temperature using HITACHI F4000 (Japan) spectrofluorimeter.

**Molecular Docking of PS Polar Head in CT II and CT 4**. Molecular docking simulations using GOLD 2.0 software [24] were performed to identify PS binding sites on the surface of CT molecules.

In our simulations, we used a crystallographic model of the 3D structure of CT А3 from *N. atra* venom (PDB code: 2BHI) [17] and NMR models of CT II in hydrated state (PDB code: 1CB9, models 1, 14 and 18) [25] and in the presence of dodecylphosphocholine (DPC) micelles (PDB code: 1FFJ, models: 1 and 10) [4].

In order to account for the receptor’s conformational mobility, we used a series of 3D models representing the dynamic behavior of the toxins in media of different polarities. Starting from experimental toxin models, we obtained representative sets of corresponding 3D models of CT 4 and CT II by molecular dynamics simulations in aqueous media and by Monte Carlo conformational search for finding low–energy states in the presence of the implicit membrane (data not published).

**Fig. 1:**
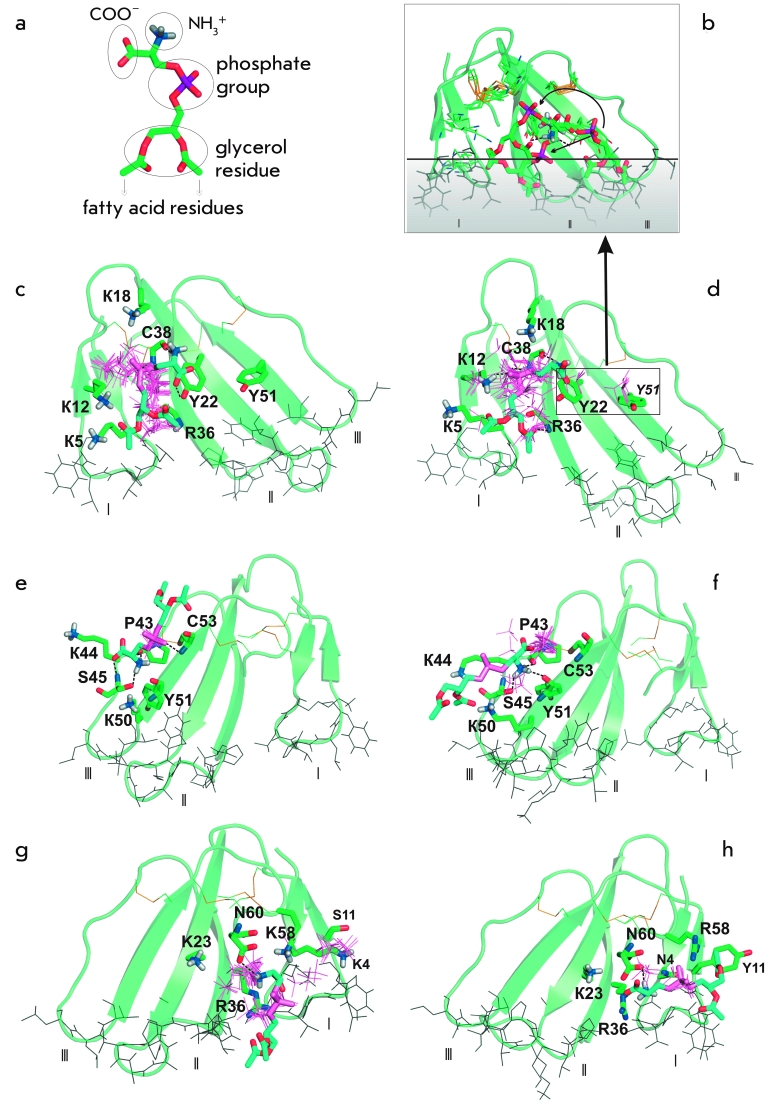
(a) 3D model of the PS polar head (hPS) used in molecular dock- ing simulations. Functional groups are indicated. (b–h): Molecular docking solutions for hPS bound in the M site of (b, d) CT 4 and (c) CT II, in the L3 site of (e) CT II and (f) CT 4, and in the L1 site of (g) CT II and (h) CT 4. 3D models of the toxins are shown in a ribbon representation. The membrane binding hydrophobic residues of loops I–III (designated with roman numbers), backbone and/or side chain atoms of the site-forming residues, and one of the preferred conformations of hPS in the related sites are displayed as stick diagrams. To demonstrate the diversity of hPS positions in the sites, the corresponding locations of the hPS phosphate group are also shown in stick representation for other docking solutions from the TOP10 set. Amino acid residues forming the sites are marked with a one-letter code, with a residue number and a standard color- ing scheme for the atom types. The phosphate group and carbon atoms of hPS are pink and cyan, respectively. (b) Different positions of hPS in the site M_Y51 of CT 4 found among the TOP10 docking solutions. A hypotheti- cal migration path of hPS from the M_ Y51 site towards the M site is shown by arrows. The phospholipid bilayer is depicted as the shadow region under the horizontal line. The location of CT 4 relative to the membrane’s interface (the horizontal line) corresponds to the proposed mode of CT binding to the lipid membrane.

The phosphatidylserine polar head (hPS) used in the simulations included all atoms of this lipid’s polar head group with СН_3 _groups, instead of fatty acid residues (Fig. 1A). hPS docking simulations were performed for 16 and 15 spatial models of CT 4 and CT II, respectively.

For each CT model, 50 complexes (docking solutions) were obtained, among which 10 with the best “gold score” criterion [26] (TOP10) were used for further analysis. Thus, for CT 4 and CT II, 160 and 150 ТОР10 solutions in total were selected, respectively. We made decisions about hPS binding sites at CT by analyzing the localization of hPS on the toxins molecule’s surface and the frequency of occurrence of corresponding complexes in the ТОР10 solutions. The hPS surface is polar; therefore, hydrogen bonds and electrostatic forces contribute most to the lipid–toxin complex energy. The docking solution sets were analyzed with respect to the number and distribution of hydrogen bonds and ionic contacts (those with distances between ionic groups below 6 Å ) in order to identify the key residues involved in the interaction with hPS in most of the complexes.

The presence of hydrogen bonds and ionic interactions was estimated using the PLATINUM [27] and GROMACS 3.3.1 [28] software packages.

## RESULTS

**Fluorescence Measurements: CT-Induced Release of Dye from Liposomes**.

**Fig. 2:**
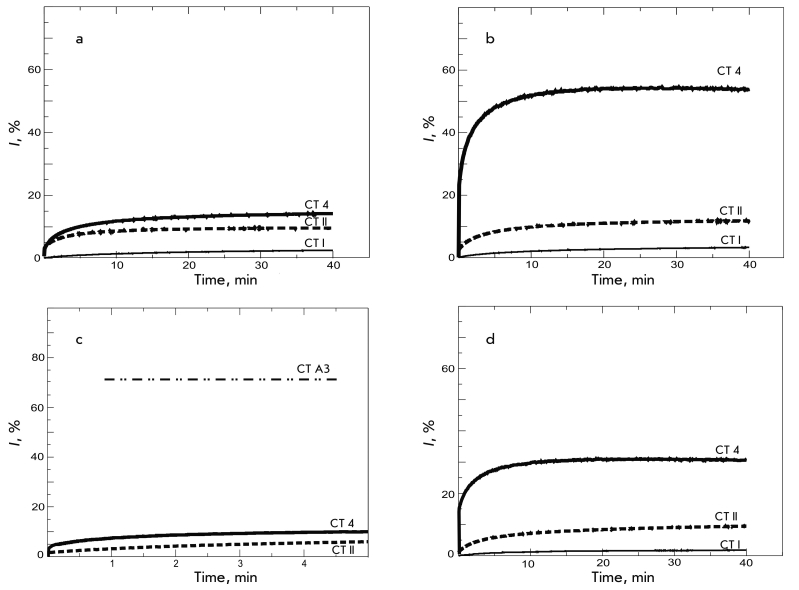
CT-induced release of fluorescent dye calcein from liposomes of different compositions: (a) pure POPC; (b) POPC/PS5%, and (d) POPC/SGC5% after addition of CT I, CT II (Naja oxiana), and CT 4 (Naja kaouthia). (c) Kinetics of 6-CF fluorescence during the initial few minutes after addition of CT II and CT 4. For this measurement, (c) the experimental conditions, such as buffer composi- tion, fluorophore and lipid/protein concentrations (10/0.16 µM) in the sample correspond to those in [6]. The data for 6-CF release induced by CT A3 (Naja atra) reported in [6] are shown as (_ .. _).

**Dependence of CT activity on a type of minor (5%) anionic lipid (PS, SGC)**. In this work, we estimated the lytic activity of P– and S–type toxins, namely CT II ( * N. oxiana *; ), CT 4 ( * N. ka* *outhia* ), and CT I ( * N. oxiana * ) by the amount of organic dye calcein released from POPC, POPC/PS5%, and POPC/SGC5% liposomes. CT I (S–type) shows almost no activity with respect to both neutral liposomes (POPC) and those containing minor quantities of anionic lipids (PS, SGC) (Fig. 2): in all cases intensity of the released calcein, * I * , was below 5%. In experiments with CT II (Р–type), the release intensity of dye was higher (compared to the results obtained with CT I), average * I * values being below 10% regardless of the liposome composition (Fig. 2).

Fluorescence measurements showed that CT 4 had the strongest damaging effect on all the liposome types studied (Fig. 2). With this toxin, a clear trend is visible: its activity depends on the presence and type of the anionic component in the liposome. Thus, the value of * I * for neutral liposomes was only ~10%, whereas for SGC–containing vesicles it was 30%. The highest CT 4 activity was measured for POPC/PS5% liposomes, with dye release reaching 50%. It should be noted that there was also a significant difference in the activity of individual toxins with respect to the anionic lipids in liposomes. For instance, CT II does not differentiate between PS– and SGC–containing liposomes, while the lytic activity of CT 4 with respect to POPC/PS vesicles is almost twice as high as that with respect to SGC–containing ones (Figs. 2B, D).

It is interesting that we did not find a specific interaction between SGC molecules and CT 4 (which is identical to CT А3 from *N. atra* venom). Moreover, when we reproduced the experimental conditions described in [6] (see the Experiment section), the value of * I * after a few minutes of incubation was less than 10%, unlike the >70% reported in [6] (Fig. 2C). The authors of [6] used 6–CF fluorofore, which, due to its lower molecular charge, is prone to spontaneously releasing from liposomes (A.V. Feofanov, private communication).

**Fig. 3:**
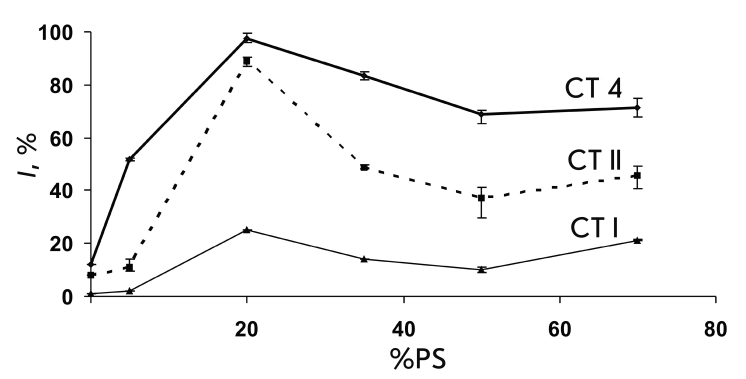
Dependence of the CT-induced calcein release, I (%), on the PS content (in %) in the POPC liposomes. The fluorescence measurements were performed 10 min after the addition of the toxins. The values of I are calculated (see the Experiment sec- tion) and averaged for 2–3 measurements.

**CT–induced permeability of POPC liposomes with different PS content. ** We measured * I * for the interaction of CT I, CT II, and CT 4 with POPC liposomes containing varying amounts of PS (20, 35, 50, and 70%) and studied the dependence of the lytic efficiency on the anionic lipid concentration. The latter turned out to be similar for all three toxins; in particular, there was a maximum in *I * at 20% PS (Fig. 3) for all CTs. The toxicity trend CT 4 >; CT II >; CT I stays the same for all liposome types at the given lipid/protein ratio (L/P = 100). The lytic effect of CT 4 and, especially, CT II increases drastically for liposomes POPC/PS20% (over 90 and 70%, respectively). At lower (5%) and higher (35% and more) PS contents, the drop in the activity curve * I * is more pronounced for CT II than for CT 4. CT I shows low lytic activity in the entire range of PS contents in the liposomes, with a weak maximum at PS20% (Fig. 3).

To explain the observed effects, we assumed a uniform distribution of PS in the liposomes and calculated the number of PS molecules per toxin molecule in liposomes of various composition that were 100 0 Å in diameter . It appeared that the number of PS molecules for liposomes, with respect to which CTs show the maximum activity, is the same as the number of potential binding sites for the lipid head found in molecular docking simulations. In the case of complete toxin binding at L/P = 100, the liposome charge is compensated in the POPC/PS20% liposomes. Thus, there are 10–12 PS molecules (on average, 3.5 PS at the toxin’s binding site) per each toxin molecule. There are 50–60 lipids in total per each toxin, and 16–18 lipids are located in direct proximity to the contact region with toxin.

For comparison, in the POPC/PS5% liposomes, charge compensation occurs at very low CT concentrations (L/P ~300); therefore, at a higher toxin content (L/P = 100), some CT molecules may not bind to the membrane. However, even if there is complete CT binding to the POPC/PS5% liposome surface, there may not be a single PS molecule at the toxin/lipid contact region (on average, there are 0.8 PS molecules per surface area in contact with a toxin molecule).

**Molecular Docking**. As a result of molecular docking simulations of hPS into 3D models of CT 4 and CT II, three hPS binding sites (referred to further as М, L3, and L1) consisting mainly of conservative residues were found on the surfaces of both toxins. One feature of the complexes that were formed is the multivariant positioning of the ligand in the sites (Fig. 1), which is due to the presence of several donor–acceptor groups capable of forming hydrogen bonds in both the ligand and the receptor.

**Fig. 4:**
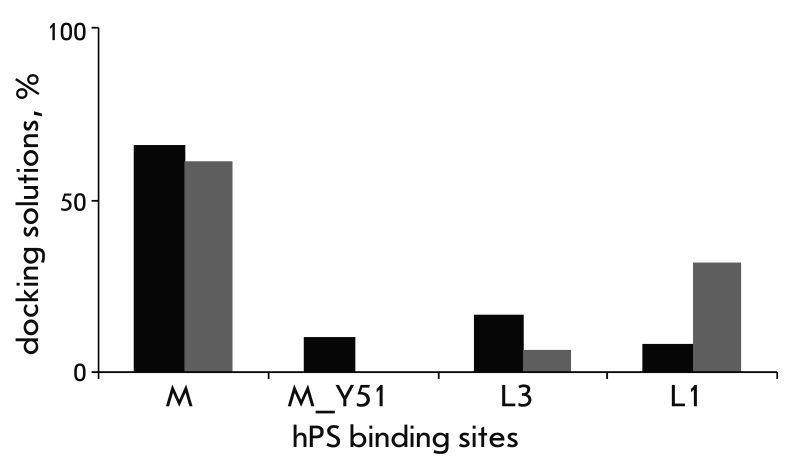
Distribution histogram of docking solutions for the hPS binding sites (М, М_Y51, L3, L1) in CT II (grey) and CT 4 (black) molecules. The best scoring solutions (TOP10) for both toxins are presented.

**The M site. ** The M site is the most frequently occurring one among the ТОР10 docking solutions; it is present in 75 and 60% of solutions for CT 4 and CT II, respectively (Fig. 4). The site M includes a “lysine” cluster (K5, K12, K18, K35) surrounding the polar surface (the site’s bed), which is formed by the backbone atoms of the residues L6, R36, G37, C38 and Y22 OH–group (Figs. 1C, D). In more than 60% of solutions for both toxins, there are three or more hydrogen bonds between key residues of the site М (K12, Y22, R36, and C38) and hPS and, usually, several ionic contacts.

The mobility of side chains of lysine residues makes site M compatible with different hPS conformations (Figs. 1C, D). This site is present in the ТОР10 docking solutions for most CT 4 and CT II models, including the toxin structures determined experimentally.

In contrast to CT II, in the ТОР10 set for CT 4, there is a group of solutions (further referred to as М_Y51) in which the Y51 residue’s hydroxyl group is involved in binding of the lipid head (Figs. 1B, 2D). In most М_Y51 solutions (10 out of 16 complexes), the hPS tail is oriented towards the tip of loop III (Fig. 1B). In the other М_Y51 solutions, the hPS glycerine group is oriented towards loop I with fewer hydrogen bonds with the ligand. Therefore, in this group of solutions, the ligand’s position is intermediate between those in the M_Y51 and М sites. In this case, Y22 is a key residue with which the ligand interacts ( * via * the hydrogen bond) during the supposed migration from the М_Y51 to the М site (Fig. 1B).

**The L3 and L1 sites.** ** In addition to the major (in the number of solutions) site М, there are two sites in the ТОР10 set, L1 and L3, located on the concave side of the CT molecule surface near loops I and III, respectively. The frequency at which these sites occur among the TOP10 docking solutions is different for CT II and CT 4 (Fig. 4).

The L3 site is formed by the bakbone atoms of the P43, Y51, C53, and S45 conservative residues, as well as by the side chain of the S45 residue. In most of the CT–hPS complexes, P43 and Y51 carbonyl groups and the S45 ОН–group fix the position of the ligand’s NH_3_^+ ^group by forming a hydrogen bond (Figs. 1E, 1). In some solutions, the hPS glycerine residue is oriented towards the tips of loops I–III and the K44 and K50 side chains are near the hPS phosphate group (Fig. 1F).

The site L1 and CT 4/CT II represents a region with high electrostatic potential containing side chains of the N4/K4, K23, R36, R58/K58, and N60 residues, which form favorable electrostatic contacts with hPS. In most docking solutions obtained for CT II and CT, the hPS NH_3_^+ ^group is located near the N60 residue, forming a hydrogen bond with the side chain carboxyl or carbonyl group (Figs. 1G, 1H). The hPS molecule accommodates various orientations with respect to the toxin molecule and suggested bilayer, including those corresponding to the lipid position in the bilayer (Fig. 1G).

## DISCUSSION

The functional activity of cytotoxins arises from their interaction with the cell membrane; however, the mechanism of this interaction at the molecular level is still unknown. The oligomerisation of CTs necessary for forming pores in the membrane [5, 6] takes place not in solution, but apparently in the vicinity of the membrane; the details of this process and the geometry of the supramolecular complex are unclear. Understanding the initial stages of the toxin–membrane interaction is extremely important for understanding the entire mechanism of cytotoxic activity.

The data obtained in this study on the release of calcein from the POPC, POPC/PS5%, and POPC/SGC5% liposomes, depending on the CT type (Р– or S–), correlate with the hypothesis that the properties of the membrane–binding motif (tips of loops I–III) influence the CT lytic activity with respect to the cell membranes. P–type CTs are known to more actively interact with the bilayer [2, 29, 30]. Indeed, both of the Р–type CTs studied, CT II and CT 4, demonstrated more pronounced lipolytic behavior (on POPC/PS5% and POPC/SGC5%) than CT I, for which intensity of the released dye did not exceed 5%. It has been suggested that, due to the presence of polar residues in loop II (S28 and D29 in CT I) of the S–type CTs, their interaction with the bilayer involves fewer residues, primarily those of loop I [2, 31]. For identical bilayer embedding geometry, the calculated free energy of binding is higher for CT I than for CT II [30].

The experiments also showed that all the toxins studied were most active with respect to PS–containing liposomal membranes, and that the dye–release dependencies on the PS content were of dome shape and with the highest lysis intensity for the POPC/PS20% liposomes. An estimation of the number of PS molecules in 1000– Å diameter liposomes at L/P = 100 suggests that the lysis intensity peak can be reached when there are no less than three PS molecules on the surface of the bilayer in contact with the toxin during embedding. Under such conditions, the difference in the intensity of the released dye for CT 4 and the significantly less active CT II is minimal (90 and ~70%) compared to the case of liposomes with a PS content other than 20%. We can speculate that the optimum PS content for lysis that ensures the involvement of all three toxin sites is very important for the efficiency of toxin–membrane interaction. The weak response observed for the nearly inactive CT I is, perhaps, due to the fact that that this toxin does not embed deep enough into the bilayer and/or because of the different binding geometry involving only one or two loops, resulting in a lower availability of sites for binding with PS.

The drastic decrease of the POPC/PS5% lysis efficiency indicates that the mechanism that binds the highly basic toxin with the anionic membrane includes nonspecific electrostatic attraction. As a result, due to the low membrane charge density and too few PS molecules as potential ligands (less than one per toxin–membrane contact region), the CT does not embed into the bilayer efficiently.

The observed decrease in CT 4 and CT II activity with respect to liposomes with PS contents of 35% and higher is most likely caused by a change in the bilayer’s properties, in particular, by an increase in the lipid packing density near the interface. For instance, the formation of mini–clusters of PS molecules becomes more probable. According to the data of molecular dynamics simulations for bilayers containing 20 and 50% PS, in the latter case there are twice as many hydrogen bonds between PS polar heads near the interface (data not published).

The estimated number of PS molecules in the POPC/PS20% liposomes interacting with CTs is the same as the number of the toxin PS–binding sites predicted by molecular docking simulations. It should be also noted that the embedding of the hydrophobic tips of loops I–III of the toxins studied into the lipid bilayer has been confirmed by NMR data on CT II [4] and CT А3 ( *N. atra* ) [6] in micelles.

The M site (residues K5, K12, K18, Y22, R36, and C38), presents in most docking solutions, is identical to the sulphatide–binding pocket located on the convex side of the toxin molecule [17]. Indeed, the results of a chemical modification of lysine and tyrosine residues suggest that the site’s key residues, such as Y22 and K12, significantly influence the functional activity of CTs [32, 33]. Judging by the number of intermolecular hydrogen bonds and ionic contacts in the docking solutions, the M site has the best potential for forming a stable complex with the ligand. Formation of the CT A3–SGC complex in of a membrane–mimicking environment [17] indicates that other anionic components in the membrane can bind to the M site. At the same time, the superimposition of CT docking models, according to the mode by which they are embedded into the bilayer, indicates different availabilities of the sites for the PS head. For instance, the M site residues do not directly interact with the bilayer. In order to reach the M site, the lipid molecule must overcome the energy barrier so that the acyl part of the lipid will penetrate the polar interfacial zone.

It is interesting that, in the М_Y51 docking models for CT 4, the lipid binding site assumes a hydrogen bond between the lipid NH_3_^+ ^group and ОН groups of both tyrosine residues, Y22 and Y51, the phosphate group being near the membrane’s surface. Therefore, we can expect that lipids reach the М_Y51 site faster than they reach the M site. Moreover, the existence of M_Y51 solutions differing in the involvement of the M site’s key residues and the orientation of the lipid’s glycerin residue can indicate a possible migration path of the lipid head from the М_Y51 site to the M site.

There is no direct experimental evidence of the existence of the L3 and L1 sites revealed by docking simulations. On the other hand, the L3 site located near loop III has a structure similar to that of the M site: the backbone atoms of this site’s residues (P43, Y51, and C53) form hydrogen bonds with the lipid’s polar head, which is additionally supported by an electrostatic interaction with the ionic groups of lysine residues (K44 and K50). Recently, a consensus sequence L/PKSSLL based on a peptide sequence alignment (related to antibodies neutralizing * N. atra * venom) revealed by phage display has been proposed as an epitope involved in the action of CT A3 from * N. atra* venom [34]. This epitope corresponds to a fragment of the loop III sequence and contains the L3 site residues (P43–S45). The L3 site is the least available for PS in the bilayer, taking into account the CT–membrane binding mode. It is possible that, during later stages of embedding, when CT penetrates deeper and this site becomes more available, binding an additional lipid strengthens the CT–membrane interaction.

In contrast, among docking solutions for the L1 site (a polar cluster comprised mainly of charged residues at the base of loops I and II) there are models of complexes with hPS in the membrane–water interfacial zone (in case CT loops embed into the membrane). This indicates the availability of this site for the bilayer hPS. There have been reports of NMR studies showing that some L1 site residues take part in dATP binding [16].

**Fig. 5:**
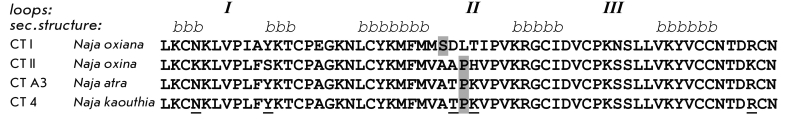
Sequence alignment for CT I, CT II, CT 4, and CT A3. The β-strands are indicated by b. Amino acid substitutions between CT A3 and CT 4 are underlined.

It is interesting to note that just five amino acid substitutions turn CT II into the much more lytically active CT 4 (Fig. 5); the total charge of the molecule (+9) is the same for both CTs. It is not surprising therefore that, along with a high sequence homology and similar 3D structure, the docking results for the two CTs are also very similar, differing only in the frequency at which the sites occur among the docking solutions. Four out of five amino acid substitutions are localized near the L1 site (more frequently in the case of CT II), and three of them—in the areas most important for the toxin—are in loops I and II. Thus, the positive charge in CT II (K4/N4 in CT II/CT 4) is transferred to loop II (H31/K31 in CT II/CT 4) in CT 4. On the one hand, this disturbs the hydrophobic pattern of loop II; on the other hand, lysine residues are known to anchor at the interface by interacting with the lipid phosphate groups [35], which may reduce the protein dissociation constant. A correlation has been found between the presence of a positive charge at the tip of loop II and CT hemolytic activity [36]. Although the K4/N4 and K58/R58 substitutions in toxins are localized relatively far from the membrane surface (assuming that the CT embeds with the tips of loops I–III), both residues are parts of the L1 site and form a hydrogen bond and/or ionic contact with the lipid’s serine head in the docking solutions. The S11/Y11 substitution increases the hydrophobicity of the CT 4’s loop I by enabling the coupling of the F10 and Y11 aromatic residues, which can act as a good hydrophobic anchor. We can speculate that, due to the less favorable binding conditions in the M_Y51 site (manifested by the absence of this site among the ТОР10 solutions for CT II), the availability of the M site for the anionic lipid head is less for CT II. The local conformational differences between CT II and CT 4 can also contribute to the rearrangement of the hPS binding locations for the L1 and L3 sites. At the same time, it is obvious that the simulation data are not sufficient for a qualitative estimation of the possible changes of the lipid binding constants for the predicted sites and, therefore, for a detailed explanation of the differences in the CT II and CT 4 activities.

Also, we would like to note that our experiments on the release of calcein from the POPC/SGC5% liposomes and reproducing the experimental conditions described in [6], where 6–CF was used as fluorophore, did not reveal any high affinity of CTs (in particular, CT 4) with respect to SGC, which was in disagreement with the data published in [6]. At the same time, the existence of the X–ray CT A3–SGC complex confirms that various anionic components in the membrane can bind to the M site, and experiments with SGC–specific antibodies and enzymes reveal a dependence between the CT A3 functional activity and the presence of SGC in the membrane [17]. This glycolipid is present in low concentration in the outer membrane leaflet of rat cardiomyocytes, hence the big interest in SGC as a potential CT target. The low reproducibility of the results achieved on model membranes, in addition to the importance of technical details (e.g., the nature of dye), emphasizes the need to exercise caution when extrapolating to cells the results obtained using model systems. The functional activity of a toxin is likely to arise due to a number of factors that are not at all limited to the affinity to various membrane components.

In conclusion, based on the data on fluorescence intensity of a due released from anionic liposomes with a varying content of an anionic lipid as a measure of CT 4, CT I, and CT II activity, as well as the results of molecular docking of hPS into CT 4 and CT II, our hypothesis is that there are specific lipid binding sites of various affinities on the toxin surface. The concept of a CT binding to the membrane surface in two stages (initially via electrostatic and then * via * hydrophobic interaction), specific complexes of toxin with the anionic lipid’s polar heads being the determinants of the process, provides qualitative insight into the dependence that the fluorescent dye release has on the PS content in the POPC liposomes.

## ABBREVIATIONS

CT: cytotoxin
PS: phosphatidylserine
PKC: protein kinase C
SGC: sulphatide
POPC: palmitoyl oleyl phosphatidylcholine
6-CF: 6-carboxyfluorescein
L/P: lipid/protein ratio
DPC: dodecylphosphocholin
NMR: nuclear magnetic resonance
hPS: phosphatidylserine polar head
PDB: Protein Data Bank

## References

[1] Dufton M.J., Hider R.C. (1988). Pharmacol. Ther..

[2] Chien K.Y., Chiang C.M., Hseu Y.C. (1994). J. Biol. Chem..

[3] Dauplais M., Neumann J.M., Pinkasfeld S. (1995). Eur. J. Biochem..

[4] Dubovskii P.V., Dementieva D.V., Bocharov E.V. (2001). J. Mol. Biol..

[5] Forouhar F., Huang W.N., Liu J.H. (2003). J. Biol. Chem..

[6] Tjong S.C., Wu P.L., Wang C.M. (2007). Biochemistry.

[7] Kumar T., Jayaraman G., Lee C. (1997). J. Biomol. Struct. & Dyn..

[8] Wang C.H., Wu W. (2005). FEBS Lett..

[9] Feofanov A.V., Sharonov G.V., Astapova M.V. (2005). J. Biochem..

[10] Chiou S.H., Raynor R.L., Zheng B. (1993). Biochemistry.

[11] Raynor R.L., Zheng B., Kuo J.F. (1991). J. Biol. Chem..

[12] Wu P.L., Lee S.C., Chuang C.C. (2006). J. Biol. Chem..

[13] Lee S. C., Guan H. H., Wang C. H. (2005). J. Biol. Chem..

[14] Sue S. C., Brisson J. R., Tjong S. C. (2001). Biochemistry.

[15] Tjong S.C., Chen T.S., Huang W.N. (2007). Biochemistry.

[16] Jayaraman G., Krishnaswamy T., Kumar S. (1999). J. Biol. Chem..

[17] Wang C.H., Liu J.H., Lee S.C. (2006). J. Biol. Chem..

[18] Dufourcq J., Faucon J.F., Bernard E. (1982). Toxicon..

[19] Chen K.C., Kao P.H., Lin S.R. (2007). Toxicon..

[20] Osorio e Castro V.R., Vernon L.P. (1989). Toxicon..

[21] Osorio e Castro V.R., Rogers A., Vernon L.P. (2001). J. Nat. Toxins..

[22] Feofanov A.V., Sharonov G.V., Dubinnyi M.A. (2004). Biokhimya.

[23] Kukhtina V.V., Weise K., Osipov A.V. (2000). Bioorg. Khim..

[24] Jones G., Willett P., Glen R.C. (1997). J. Mol. Biol..

[25] Dementieva D.V., Bocharov E.V., Arseniev A.S. (1999). Eur. J. Biochem..

[26] Jones G., Willett P., Glen R.C. (1995). J. Mol. Biol..

[27] Pyrkov T.V., Chugunov A.O., Krylov N.A. (2009). Bioinformatics.

[28] Lindahl E., Hess B., van der Spoel D. (2001). J. Mol. Model..

[29] Dubovskii P.V., Lesovoy D.M., Dubinnyi M.A. (2003). Eur. J. Biochem..

[30] Dubovskii P.V., Lesovoy D.M., Dubinnyi M.A. (2005). J. Biochem..

[31] Efremov R.G., Volynsky P.E., Nolde D.E. (2002). J. Biophys..

[32] Gatineau E., Toma F., Montenay-Garestier T. (1987). Biochemistry.

[33] Gatineau E., Takechi M., Bouet F. (1990). Biochemistry.

[34] Wang P.C., Loh K.S., Lin S.T. (2009). Biochem. Biophys. Res. Commun..

[35] Désormeaux A., Laroche G., Bougis P.E. (1992). Biochemistry.

[36] Hider R.C., Khader F. (1982). Toxicon..

